# Comparative analyses of alphaviral RNA:Protein complexes reveals conserved host-pathogen interactions

**DOI:** 10.1371/journal.pone.0238254

**Published:** 2020-08-25

**Authors:** Natasha N. Gebhart, Richard W. Hardy, Kevin J. Sokoloski

**Affiliations:** 1 Department of Molecular and Cellular Biochemistry, College of Arts and Sciences, Indiana University, Bloomington, Indiana, United States of America; 2 Department of Biology, College of Arts and Sciences, Indiana University, Bloomington, Indiana, United States of America; 3 Department of Microbiology and Immunology, University of Louisville School of Medicine, Louisville, Kentucky, United States of America; 4 The Center for Predictive Medicine for Biodefense and Emerging Infectious Diseases, University of Louisville School of Medicine, Louisville, Kentucky, United States of America; CEA, FRANCE

## Abstract

The identification of host / pathogen interactions is essential to both understanding the molecular biology of infection and developing rational intervention strategies to overcome disease. Alphaviruses, such as Sindbis virus, Chikungunya virus, and Venezuelan Equine Encephalitis virus are medically relevant positive-sense RNA viruses. As such, they must interface with the host machinery to complete their infectious lifecycles. Nonetheless, exhaustive RNA:Protein interaction discovery approaches have not been reported for any alphavirus species. Thus, the breadth and evolutionary conservation of host interactions on alphaviral RNA function remains a critical gap in the field. Herein we describe the application of the Cross-Link Assisted mRNP Purification (CLAMP) strategy to identify conserved alphaviral interactions. Through comparative analyses, conserved alphaviral host / pathogen interactions were identified. Approximately 100 unique host proteins were identified as a result of these analyses. Ontological assessments reveal enriched Molecular Functions and Biological Processes relevant to alphaviral infection. Specifically, as anticipated, Poly(A) RNA Binding proteins are significantly enriched in virus specific CLAMP data sets. Moreover, host proteins involved in the regulation of mRNA stability, proteasome mediated degradation, and a number of 14-3-3 proteins were identified. Importantly, these data expand the understanding of alphaviral host / pathogen interactions by identifying conserved interactants.

## Introduction

The members of the genus *Alphavirus* are single stranded positive-sense RNA viruses, many of which represent significant concerns to public health [1]. Alphaviruses are broadly classified into one of two groups on the basis of disease symptomology. Members of the arthritic group, such as Sindbis virus (SINV) [2–5]; Ross River virus [6–9]; and Chikunguya virus (CHIKV) [10–14], are capable of causing severe mutli-joint arthritis in otherwise healthy individuals [15]. In contrast, members of the encephalitic group, such as Venezuelan Equine Encephalitis virus (VEEV); and Western or Eastern Equine Encephalitis viruses, cause viral encephalitis, predominantly in young children and the elderly [16–20].

Despite the recognized potential to cause large scale outbreaks of severe disease, there are no FDA approved vaccines or specific antiviral therapies to prevent or treat alphaviral disease. An underlying cause of the lack of broad-acting anti-alphaviral therapies is the considerable gap of knowledge in regards to alphaviral RNA host / pathogen interactions. While host / pathogen interactions have been studied in medically relevant and model alphaviruses, research efforts have primarily focused on identifying the Protein:Protein interactions of the nonstructural proteins or replication complexes [21–25]. While these efforts have led to several notable discoveries, the extent to which host factors interact with the alphaviral viral RNAs (vRNAs) during infection have to date been overlooked by host / pathogen interaction discovery approaches despite synonymous characterizations of flaviviruses [26].

Nonetheless, instances of vRNA:Protein interactions have been reported for several alphaviruses. Examples include the identification of interactions between the cellular La protein and the vRNAs in the mammalian and mosquito hosts [27–29]. In addition the cellular hnRNP K protein has been identified as a key regulator of the subgenomic RNA of SINV [30, 31]. Another hnRNP protein, hnRNP A1 interacts with the SINV minus strand RNA to enhance promoter selection [32, 33]. In addition, the cellular HuR protein interacts with the SINV 3’UTR to stabilize the vRNAs during vertebrate and invertebrate infections [34, 35].

Recently, we reported the development and application of an innovative discovery approach capable of identifying host / pathogen interactions in an unbiased manner during bona fide viral infection [31]. This strategy, termed the Cross-Link Assisted mRNP Purification (CLAMP) assay, was used to identify interactions between the vRNAs of SINV and proteins of the host cell. Importantly, this work led to the identification and characterization of novel SINV vRNA:Protein interactions. An exceptional strength of this approach is its capacity to be readily adapted to other viruses without genetic modification of the host or pathogen. As such, here we report the use of the CLAMP assay to identify the Protein:vRNA interactions of CHIKV and VEEV. Through the use of comparative analyses we further identify a series of conserved alphaviral host / pathogen interactions which may be exploited in future research; and ontological assessments reveal cellular pathways which may be ready targets for the development of broad-spectrum anti-alphaviral compounds.

## Materials and methods

### Tissue culture cells, viruses, and other relevant molecules

BHK-21 and 293HEK cells (gifts from the lab of Dr. Charles Rice) were cultured in whole growth medium consisting of minimal essential media (MEM, Cellgro), 10% Fetal Bovine Serum (Atlanta Biologicals), 1x nonessential amino acids (Cellgro), 1X antibiotic / antimycotic solution (Cellgro), and L-glutamine (Cellgro). All cells were incubated in a humidified incubator with an atmosphere of 5% CO_2_ at 37°C.

All viruses were prepared from infectious cDNA clones as previously described [31]. Briefly, SINV strain Toto1101, CHIKV strain 181/25, and VEEV strain TC-83 were *in vitro* transcribed to yield infectious genomic RNAs. Approximately 10μg of RNA was electroporated into actively growing BHK-21 cells. After the development of significant cytopathic effects, the cell supernatants were harvested and clarified via centrifugation at 8,000xg for 10 minutes. The resulting P(0) stocks were aliquoted into small volume samples, and assayed by standard plaque titration. Virus stocks were stored at -80°C until needed.

### The Cross-Link Assisted mRNP Purification (CLAMP) strategy

The CLAMP assay is a two-part process that results in the purification of proteins which interact with the viral RNAs in an unbiased manner. All CLAMP purifications described in this manuscript were conducted in parallel. Per sample 1x10^8^ 293HEK cells were infected at an MOI of 10 PFU/cell with either SINV (strain Toto1101), CHIKV (strain 181/25), VEEV (strain TC-83), or Mock infected via the addition of a similar volume of media. The tissue culture inoculum was aspirated, and the tissue culture monolayers were washed with 1xPBS following a one hour adsorption period. The cell monolayers were incubated under normal conditions in whole media. At 2hpi the media was replaced with warm, pre-conditioned whole media supplemented with 10μg/ml Actinomycin D. After a 15 minute incubation period (to inhibit cellular RNA synthesis), an excess volume of whole medium supplemented with Actinomycin D and 4-ThioUridine (4SU) at a concentration of 100μM was added to the tissue culture plates. After a four hour incubation period (or 6hpi) the co-transcriptional labeling media was removed and washed once with 1xPBS to remove unincorporated 4SU. The cells were then collected via gentle scraping in 1xPBS, and pelleted by centrifugation at 300xg for 5 minutes. The cell pellets were resuspended in 10mls of a 1.0% (v/v) solution of Formaldehyde in 1xPBS and cross-linked for 7 minutes under gentle rocking. Afterwards the cells were repelleted via centrifugation at 1,000xg for 3 minutes, to yield a formaldehyde cross-linking period of no longer than 10 minutes. The cross-linked cell pellets were washed with 1xPBS and resuspended in a 0.25M Glycine solution in 1xPBS. After a short incubation period the cells were pelleted as before, washed with 1xPBS and frozen at -80°C until lysate generation.

Lysates were generated from the cross-linked cells by the addition of RIPA buffer (50mM Tris HCl pH7.6 / 150mM NaCl / 1.0% (v/v) NP-40 / 0.5% (m/v) Deoxycholic acid / 0.1% (m/v) Sodium Dodecyl Sulfate (SDS)) and mechanical grinding in an aerosol-tight 15ml vitrified tissue grinder for 1 minute on ice. After grinding, the final volume of the lysate was increased to 1.25ml, and the lysates were further homogenized via repeated extrusion through a sterile 30-gauge needle. The homogenized lysates were clarified via centrifugation at 18,000xg for 5 minutes to remove insoluble materials. The clarified cross-linked lysates were subsequently transferred to a fresh microfuge tube and affinity purified using pre-loaded resin. Per sample, 500μl (packed volume) of washed streptavidin resin was saturated with an excess quantity of HPDP-Biotin ((3aS,4S,6aR)-hexahydro-2-oxo-N-[6-[[1-oxo-3-(2-pyridinyldithio)propyl]amino]hexyl]-1H-thieno[3,4-d]imidazole-4-pentanamide–Biotin) at room temperature for a period of 30 minutes. The preloaded resin was washed twice with 1xPBS to remove unbound HPDP-Biotin, followed by resuspension in RIPA buffer.

The biotinylation and capture of the vRNAs was achieved via a 1 hour incubation at 16°C under agitation. After binding, the resin was collected via centrifugation, and the unbound materials were discarded. The resin was washed once with RIPA buffer, and three times with RIPA buffer supplemented with 1M Urea. After washing the resin was exchanged into 1xPBS supplemented with 1.0% SDS via two additional washes. The formaldehyde cross-linking was reversed via heating the resin in a minimal volume of 1xPBS supplemented with 1.0% SDS at 70°C for 1 hour. The supernatants were harvested via high-speed (>20,000xg) centrifugation of the capture resin.

### Assessment of viral RNA kinetics and capture of 4SU-labeled RNAs

To assess the viral RNA abundances and synthesis kinetics of SINV, CHIKV, and VEEV 293HEK cells were infected with the aforementioned alphaviruses at an MOI of 10 PFU/Cell. After an one hour adsorption period the cells were washed, and incubated under the CLAMP assay conditions described above foregoing any RNA:Protein cross-linking. At 0.5, 1, 2, 4, and 6 hours post infection total RNA was harvested from the cells using TRIzol, as per the manufacturer’s instructions. The total RNA was then reverse transcribed using primers specific for the genomic, subgenomic, and minus strand RNAs as previously described. In addition to the viral transcripts, the host RNAse P mRNA was primed during the reverse transcription reactions to as an endogenous control to enable the normalization of the qPCR assay. Absolute viral copy numbers were determined by comparing the Ct values of the strand specific cDNAs to standard curves of known abundance, as previously described [31].

To determine the efficiency with which the 4SU-labeled viral RNA species are purified during the CLAMP assay, cross-linked RNA:Protein complexes were extracted, biotinylated and purified as described above. However, rather than releasing and harvesting the retained proteins for mass spectrometry analyses, the RNAs purified by the CLAMP process were extracted using TRIzol reagent after the reversal of the RNA:Protein cross-linking after elution of the purified cross-linked RNA:Protein complexes via the de-biotinylation of the viral RNAs via addition of 50mM DTT. The purified RNAs were then used as input materials for strand specific reverse transcription reactions, as described above. The abundances of the viral RNA species, and the host RNAse P mRNA, were then detected using qRT-PCR to determine which RNA species were purified during the CLAMP strategy. The individual purifications were compared relative to Input controls consisting of an equivalent volume of CLAMP extract, processed identically to that above with the exception that the streptavidin purification was skipped prior to TRIzol extraction.

### Mass spectrometric analysis of CLAMP purified proteins

The preparation of the mass spectrometric samples was performed as follows. The CLAMP purified proteins were TCA precipitated and resuspended in 100mM Ammonium Bicarbonate solution supplemented with 8M Urea. The protein pellets were vortexed until completely resuspended before the addition of Dithiothreitol (DTT) to a final concentration of 10mM. The protein samples were incubated for a period of 1 hour at room temperature prior to alkylation. Protein alkylation was achieved via the addition of freshly prepared Iodoacetamide to a final concentration of 20mM, and a 1-hour incubation at room temperature in the dark. After the alkylation period the excess Iodoacetamide was quenched via the addition of DTT to a final concentration of 40mM, after which the samples were diluted with 100mM Ammonium Bicarbonate solution to reduce the concentration of Urea to <1M. A standardized quantity (1μg) of proteomics grade trypsin was added to each sample, and the proteins were digested at 37°C overnight to generate a peptides for MS.

The tryptic libraries were analyzed by the Laboratory for Biological Mass Spectrometry at the Department of Chemistry of Indiana University- Bloomington. The peptide libraries were dried down and injected into an Eksigent HPLC apparatus coupled to an LTQ Velos mass spectrometer operating in “top eight” data-dependent tandem-MS (MS-MS) selection. The peptides were separated by a 75μm x 15cm column packed with C_18_ resin at a flow rate of 300 nl/min. A 2-hour gradient from a solution of 2% acetonitrile and 0.1% formic acid to 100% acetonitrile and 0.1% formic acid was used to resolve the peptide fragments. MS-MS peak lists were searched against the Swiss-Prot *Homo sapiens* database using Protein Prospector (v5.10.14). Carbidomethylation of cysteine residues was set as a fixed modification; and acetylation of the protein amino terminus, oxidation of methionine, and pyroglutamine formation of peptide N-terminal glutamines were set as variable modifications. A mass tolerance of 0.6 Daltons was used for precursor and fragment ions. Expectation values for the peptides was set to <0.05, but for proteins identified by a single peptide the expectation value was set to <0.01. All CLAMP mass spectrometry data sets may be found in the S1 Dataset associated with this manuscript.

### Assignment of CLAMP-identified interactants

The mass spectrometry data was used to generate lists of detected proteins for subsequent parsing to identify potential interactants. Interactants were identified through a series of comparative analyses. First, in order to be considered as an interactant a particular protein must have been detected in both independent data sets. Note that this is a relaxation of the previously reported criteria (the reasons for which are described below) [31]. Finally, in order to be considered a specific interactant the protein must not have been detected in the Mock data set. Interactant lists may be found in the S1 Table.

It is important to directly state and acknowledge that the CLAMP assays described in this report were all conducted in parallel at the same time- including those previously reported in the literature as part of an independent report [31]. In reality, the prior publication of the SINV CLAMP data set represented a small piece of a much larger series of CLAMP data sets. The decision to independently report the SINV CLAMP assay prior to these comparative analyses was based largely on technical expediency and resource limitations. Thus, all comparisons made amongst the individual CLAMP data sets as described in this study are appropriate and benefit from being generated and assessed in parallel in regards to their infections, purifications, and mass spectrometric analyses.

### Ontological analysis of CLAMP-identified interactants

The lists of CLAMP-identified interactants for SINV, CHIKV, and VEEV were compared to those detected during the analyses of Mock infected samples to identify nonspecific interactants. Any CLAMP-interactant identified in Mock infected samples were removed from the final alphaviral lists prior to further analyses. Ontological analyses were performed using the Database for Annotation, Visualization and Integrated Discovery (DAVID, v6.8) [36, 37]. Briefly, the Uniprot accession numbers corresponding to the alphavirus specific interactants were uploaded and compared to the *Homo sapiens* background list to identify enriched Molecular Function and Biological Process ontological categories. All categories enriched above expected (as determined by the *Homo sapiens* reference genome) were considered for statistical analysis. To ensure analytical rigor, a statistical threshold of <0.05 was maintained via the examination of Benjamini corrected p-Values.

### Comparative analyses of alphaviral CLAMP-identified interactants

To identify common interactants amongst the tested alphavirus species the individual lists of CLAMP-identified interactants were sorted and compared to identify conserved Uniprot accession numbers. All possible two-pair combinations, namely- SINV/CHIKV; CHIKV/VEEV; and SINV/VEEV, were assessed. However, only the three-way comparison of SINV/CHIKV/VEEV is reported in detail in this manuscript. The paired comparisons, and their subsequent analyses, may be found in the S1 Table accompanying this manuscript.

Ontological analysis of the common alphaviral interactants was performed identically to that described in the previous subsection. In addition to the ontological enrichment analyses described above, STRING database analysis was used to identify Protein:Protein interactions amongst the common alphaviral CLAMP-interactants [38, 39]. The STRING analysis was of the CLAMP-identified alphaviral interactants considered active interaction sources obtained from gene fusion, co-occurrence, experiments, databases, and text mining, and the confidence stringency was set to Medium (0.400). STRINGs maps were generated using Cytoscape (v3.6.0) and interaction clusters were identified via k-means clustering analysis.

### Quantitative immunoprecipitation of select CLAMP identified interactants

To validate and confirm select RNA:Protein interactions 293HEK cells were infected with either SINV, CHIKV, or VEEV at an MOI of 10 PFU/Cell. The cells were treated identically to the conditions described in the CLAMP assay above, with the exception that the use of 4SU and Actinomycin D were omitted. After the solubilizing the cross-linked cells the whole cell lysates were centrifuged at 16,000xg for 5 minutes to remove insoluble materials. The clarified mixed supernatants were transferred to fresh microfuge tube, and to conserve reagents, equal volumes of SINV, CHIKV, and VEEV lysates were combined and processed together in batch form. To pre-block the batched lysates 50ul of Protein G Mag Beads (50% slurry in RIPA buffer) was added to a volume of 300ul lysate. After an hour incubation period at room temperature with gentle agitation the magnetic beads were collected, and the pre-blocked clarified batch lysates were transferred to a fresh microfuge tube. In parallel to the pre-blocking of the batched lysates, target specific antibodies were pre-bound to Protein G Mag beads. Briefly, per target 10ul of anti-hnRNP K (F45 P9 C7, Thermo Fisher Scientific), anti-hnRNP A1 (MA126736, Invitrogen), anti-ANP32a (aka PHAP I; PA5-80339 Invitrogen), or as a control anti-IL-1 (17H18L16 Invitrogen) antibodies were incubated with 50ul of Protein G Beads (50% slurry) were bound in a final volume of 100ul (diluted in RIPA buffer) for one hour at room temperature. Unbound antibodies were washed from the Protein G Mag beads, and 300ul of pre-blocked batch lysate was added to each of the pre-bound Protein G Mag beads. Binding was allowed to proceed at room temperature for one hour under gentle agitation, prior to the collection of the Mag beads and the removal and disposal of the supernatant. The RNA:Protein complexes were washed as described above, however Urea was omitted from the wash buffers to prevent the disruption of the antibody:protein complexes. After the final wash the beads were resuspended in 200ul of 1xPBS supplemented with 1.0% SDS, and the cross-linking was reversed via heating at 70°C for 1 hour. The immunoprecipitated RNAs were then extracted using TRIzol. The immunoprecipitated RNAs were then reverse transcribed with random hexamer to generate cDNAs, and assessed via qRT-PCR to detect the positive-sense viral RNAs of SINV, CHIKV, and VEEV, and the cellular 18S rRNA. Quantitative analysis of viral RNA recovery was achieved by comparing the relative quantities of the viral RNAs of each target specific immunoprecipitation with the negative control immunoprecipitations.

## Results

### Purification of alphaviral host-pathogen interactions via Cross-Link Assisted mRNP Purification (CLAMP) assay

**Fig 1:**
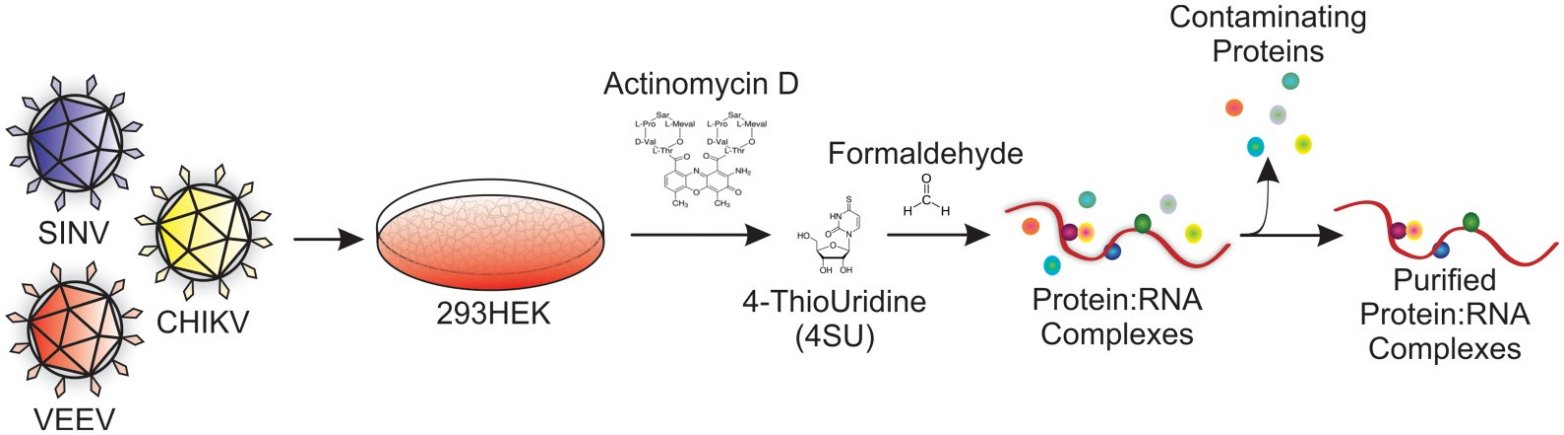
A schematic diagram of the Cross-Link Assisted mRNP Purification (CLAMP) strategy.

A primary goal of these studies was to identify host-pathogen interactions that are conserved across several medically relevant alphaviruses. To this end, we utilized a previously described novel identification approach to detect host factors that associate with the viral RNAs during infection [31]. Briefly, and as diagrammed in Fig 1, 293HEK cells were infected by either SINV, CHIKV, or VEEV, at a Multiplicity of Infection (MOI) of 10 Infectious Units (IU) per cell. Control reactions consisting of Mock infected cells were treated identically, in parallel with the above infections. After a one hour adsorption period the inoculum was removed, and the tissue culture monolayers were washed prior to the addition of growth medium. The cells were incubated under typical conditions for two hours, after which the tissue culture medium was removed and replaced with fresh medium containing Actinomycin D. After the inhibition of cellular transcription, the medium was replaced with growth medium supplemented with 4-thiouridine (4SU) to co-transcriptionally label the newly synthesized viral RNAs. The infections were allowed to proceed under normal conditions for a period of 4 hours. Subsequently, the growth medium was removed, and the cell monolayers were scraped into cold 1xPBS and harvested via centrifugation. To cross-link the genuine RNA:Protein and Protein:Protein complexes formed during infection, the cell pellet was gently resuspended in a 1% Formaldehyde solution prepared in 1xPBS [31, 40]. The cells were cross-linked for a period of no more than 10 minutes prior to quenching. The cross-linked cells were pelleted and washed twice with 1xPBS and stored at -80°C. Whole cell lysates were generated from the frozen cell pellets by tissue grinding in a minimal amount of RadioImmunoPrecipitation Assay (RIPA) Buffer. The crude extracts homogenized by several rapid passes through a 30-gauge needle, after further dilution with RIPA buffer. The extracts were clarified via centrifugation, and transferred to a fresh microfuge tube.

The individual samples were then affinity purified to enrich the cross-linked RNA:Protein complexes. Specifically, the clarified lysates described above were incubated with streptavidin resin pre-complexed with HPDP-Biotin, a biotin moiety with a reactive sulfhydryl group. The unbound materials were removed following centrifugation, and the resin was washed several times with RIPA supplemented with urea. The purified complexes were released via heating in the presence of 1.0% SDS in 1xPBS. As previously published, this method resulted in the release of highly purified RNA:Protein, and accessory Protein:Protein complexes, without releasing nonspecifically biotinylated protein contaminants. The purified materials were precipitated via TCA treatment, zip-tip exchanged, trypsin digested, and assessed via mass spectrometry.

### Analysis of RNA input and recovery during the CLAMP method

**Fig 2:**
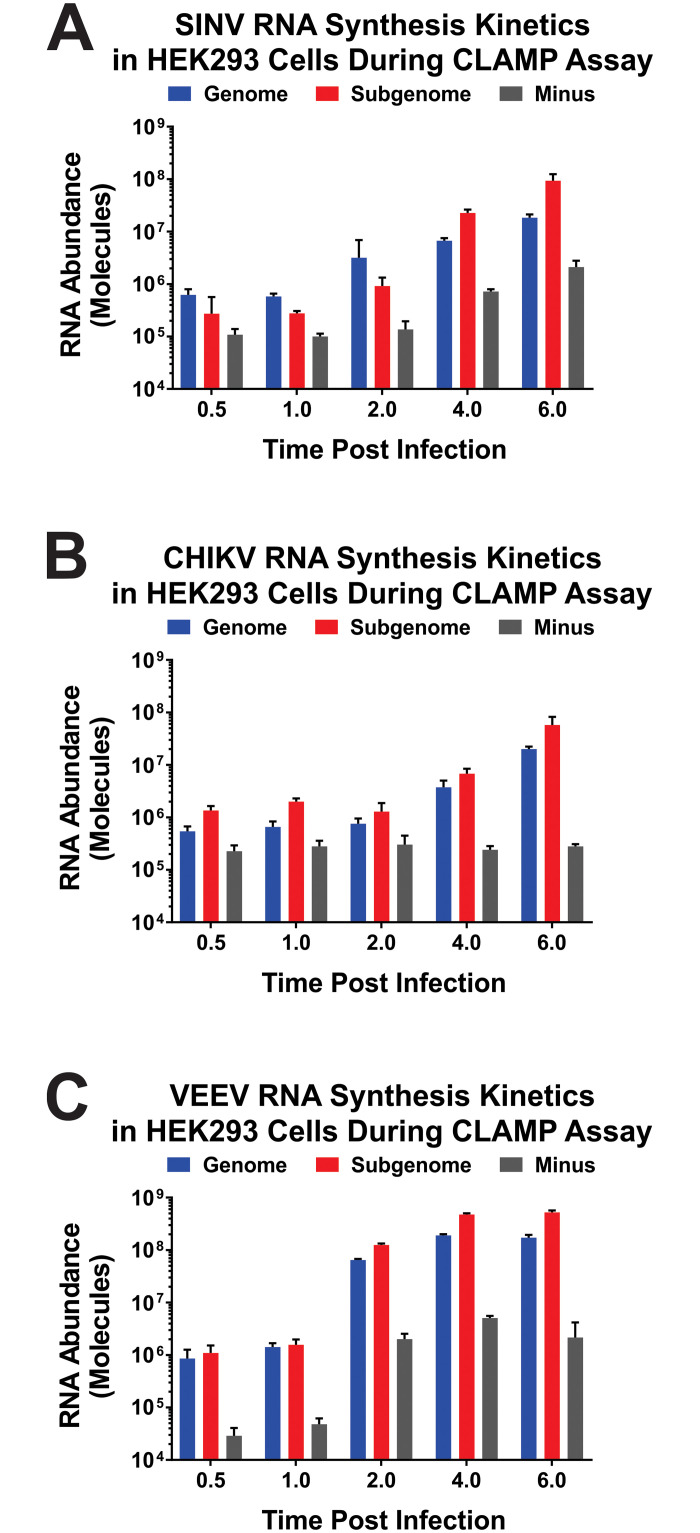
Quantitative analysis of alphaviral RNA synthesis during the CLAMP assay. 293HEK cells were infected with SINV, CHIKV, or VEEV at an MOI of 10 PFU/Cell and treated as described in the materials and methods. At the indicated times post infection total cellular RNA was extracted and quantitatively assessed using strand-specific standard curve qRT-PCR. Absolute viral RNA abundances for each of the viral RNA species, and the time post infection, are plotted on the Y- and X-axes, respectively for SINV (Panel A), CHIKV (Panel B), and VEEV (Panel C). The quantitative data shown is the means of three independent biological replicates, and the error bar represents the standard deviation of the means.

Despite SINV, CHIKV, and VEEV having highly similar molecular life cycles, each virus is known to exhibit nuances in regards to their relative replication and growth kinetics. Therefore, to confirm that the CLAMP assay described above would not be influenced by differences in viral replication kinetics, we quantitatively assessed the accumulation of the SINV, CHIKV, and VEEV viral RNA species during the time period pertinent to the CLAMP assay. Briefly, total RNA was harvested from infected 293HEK cells at regular intervals post infection, and the genomic, subgenomic, and minus strand viral RNAs were detected using strand specific standard curve qRT-PCR. As shown in Fig 2, SINV, CHIKV, and VEEV all exhibited similar replication profiles during the CLAMP assay time period. All viral RNA species accumulated with respect to time, and the rate of viral RNA synthesis is increased as viral infection proceeded. The overall RNA abundances of the viral RNA species do modestly differ across the alphaviruses, with VEEV exhibiting a higher degree of vRNA synthesis relative to either SINV or CHIKV. Interestingly, the most notable difference between the three alphavirus species is the magnitude and rate to which the Minus strand template RNA accumulated during infection. In all cases the viral RNA levels followed the standard paradigm of the abundances of the Subgenomic RNA > Genomic RNA > Minus RNA.

**Fig 3:**
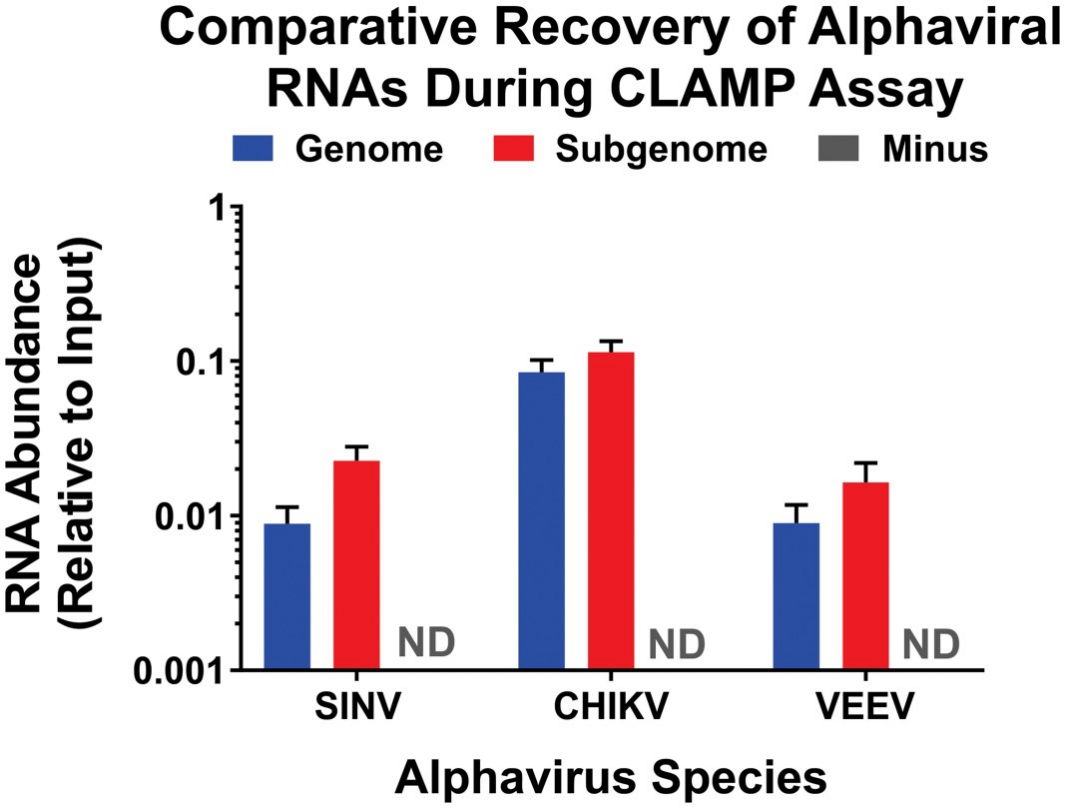
Comparative recovery of alphaviral RNAs during the CLAMP assay. As described in the materials and methods 293HEK cells were infected with SINV, CHIKV, or VEEV at an MOI of 10 PFU/Cell and co-transcriptionally labeled with 4SU during the incubation period. At the end of the incubation period whole cell lysates were generated, and the 4SU-labeled RNAs were biotinylated and purified using streptavidin resin. After the reversal of the RNA:Protein cross-links and the elution of the 4SU-labeled RNAs from the resin via cleavage of the HPDP-biotin group the purified RNAs were extracted and assessed using strand-specific qRT-PCR. The individual RNA abundances were normalized to their respective input quantities and plotted in regards to relative RNA abundance. The symbol ND indicates not detected. The quantitative data shown is the means of three independent biological replicates, and the error bar represents the standard deviation of the means.

As the overall viral RNA synthesis profiles were more or less equivalent amongst the three alphaviruses, we next sought to assess the efficiency to which the viral RNA species were purified during the CLAMP assay. To this end we performed the CLAMP assay identically to that described above for the capture of the interacting proteins, but focused instead on assessing the purified RNA species. As shown in Fig 3, the CLAMP assay resulted in the capture / purification of the positive-sense viral RNAs. Interestingly, a higher proportion of CHIKV RNA was recovered relative to the SINV and VEEV purifications. As one would reasonably expect from the viral RNA synthesis profiles, the RNA species that was primarily captured was the subgenomic RNA. In all cases we were unable to detect the Minus Strand RNA. Similarly, the cellular RNAseP gene was either undetected, or in instances where qRT-PCR amplification was observed the T_m_s of the products were indicative of nonspecific amplification.

Altogether these data indicate that the CLAMP assay conditions described in this study result in the assessment of the RNA:Protein complexes of the positive-sense RNAs of SINV, CHIKV, and VEEV. Furthermore, the interactions detected are likely to be skewed towards those of the subgenomic RNA, however complexes of the genomic RNAs are also likely to be represented in the CLAMP data sets.

### Identification of alphaviral host-pathogen interactions purified via CLAMP

Previously, we utilized a rigorous set of criteria designed to reduce or eliminate type-I errors during the analysis of SINV host pathogen interactions via CLAMP [31]. In order to be classified as a genuine CLAMP interactant, these criteria required that an individual protein be identified in both of the independent biological replicates, and be represented by no less than 3 unique peptides. Nonetheless, *a priori* the prior criteria has the potential to introduce bias by discriminating against small molecular weight, and hence, short in regards to primary sequence, proteins. Similarly, proteins that have peptide fragments outside the tolerance range of the mass spectrometer, or which poorly ionize, would skew the resulting data. Thus, we revised our initial criteria to eliminate, or reduce, bias during identification prior to the establishment of a quantitative data based triage. To this effect, we established new criteria for the assessment of CLAMP-identified host factors.

In order to eliminate unintentional bias, the CLAMP mass-spectrometric data were assessed using modified criteria. To eliminate any bias introduced against proteins which would inherently produce few peptides, to be considered a potential CLAMP-identified interactant an individual protein had to be observed in both biological replicates, regardless of the number of unique peptides observed. Collectively, this approach led, as shown in Table 1, to the identification of 449 putative interactants for SINV, 339 for CHIKV, 286 for VEEV, and 136 for MOCK. As with our previous report, we removed any proteins identified as present in the Mock samples from the viral specific CLAMP target lists. While this has the potential to introduce type-II errors, it is essential to maintaining the utility of the CLAMP approach, as normalizations of the data sets by number of unique peptides, spectral counts, or peptide coverage would be unduly influenced by variations in purification and detection. Removal of proteins identified in the Mock sample reduces the number of CLAMP-identified interactants to 355 for SINV, 256 for CHIKV, and 203 for VEEV.

**Table 1:** Summary of CLAMP identified host factors.

CLAMP Sample	Total ID’d Interactants	Specific Interactants^1^
**Mock**	136	---
**SINV**	449	355
**CHIKV**	339	256
**VEEV**	286	203

^1^ Specific Interactants are defined as those present viral samples but absent from Mock.

**Fig 4:**
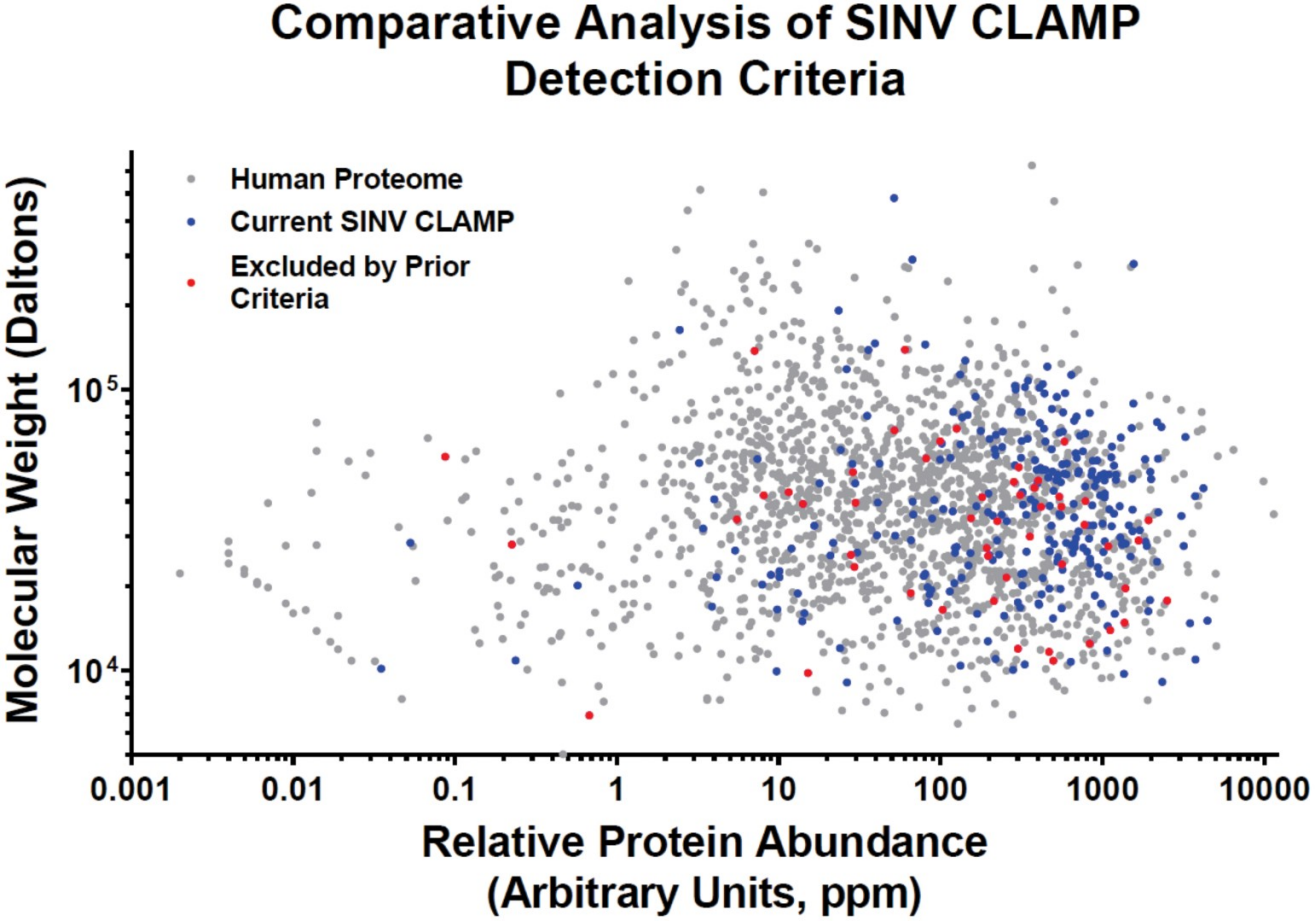
Comparative analysis of CLAMP detection criteria. The proteins of the human proteome are graphed via their respective protein abundances and molecular weight on the X- and Y-axes, respectively. SINV CLAMP interactants identified using the criteria outlined in this study are colored either Blue or Red. The individual proteins highlighted in Red are those that were excluded from the LaPointe et al., 2018 data set, based on the application of the prior criteria. Proteins of the human proteome that were undetected are shown in gray. Protein abundances were inferred from data available via the PaxDb, *Homo sapiens* integrated cell line database [41].

It should be noted that the SINV and Mock data sets analyzed in this manuscript are one in the same with those of our previous report; however, It should be noted that despite the SINV CLAMP assay being previously independently reported, all of the CLAMP assays reported in this study were all conducted at the same time in parallel. Thus, the previously published SINV and Control CLAMP assays are directly related to the CHIKV and VEEV CLAMP assays now reported here. The altering of the identification criteria, as described above, increased the number of CLAMP-identified specific interactants from 279 to 355, an increase of ~27%. Interestingly, however, analysis of the SINV CLAMP-identified specific interactants indicated that the newly included interactants spanned a wide range of molecular weights, with a mean of ~44kDa (+/- 35kDa). Nonetheless, if the newly restored CLAMP-identified interactants are assessed based on their relative protein abundance, it becomes apparent that relaxing the criteria has expanded the detection of lower abundance proteins (Fig 4). Thus, while the impetus behind altering the criteria was to prevent molecular weight bias, relaxing the criteria improved the detection of low abundance interactants. Comprehensive lists of the individual interactants identified can be found in the supplemental files (S1 Table) accompanying this manuscript.

### Ontological analyses of alphaviral CLAMP-identified interactants

As described above, the CLAMP strategy leads to the identification of host factors that were associated with the vRNA during infection. Nonetheless, as demonstrated by the data in Table 1, this discovery approach results in large data sets that must be further assessed to identify meaningful trends and themes. To parse the data sets into manageable information we used ontological analyses to describe the underlying molecular functions and biological processes of the CLAMP-identified interactants. Briefly, the CLAMP-identified interactants were assessed using the Database for Annotation, Visualization and Integrated Discovery (DAVID, v6.8) to identify the ontological categories that were enriched relative to the background human genome [36, 37]. However, since ontological analyses are prone to type-I error, the Benjamini correction was applied and a threshold of 0.05 was used to reduce the likelihood of false enrichment. As described below, ontological analyses of the CLAMP-identified interactant data sets reveals specific Molecular Function and Biological Process ontological groups are statistically enriched within each alphavirus.

#### Ontological analyses of SINV CLAMP-identified interactants

**Fig 5:**
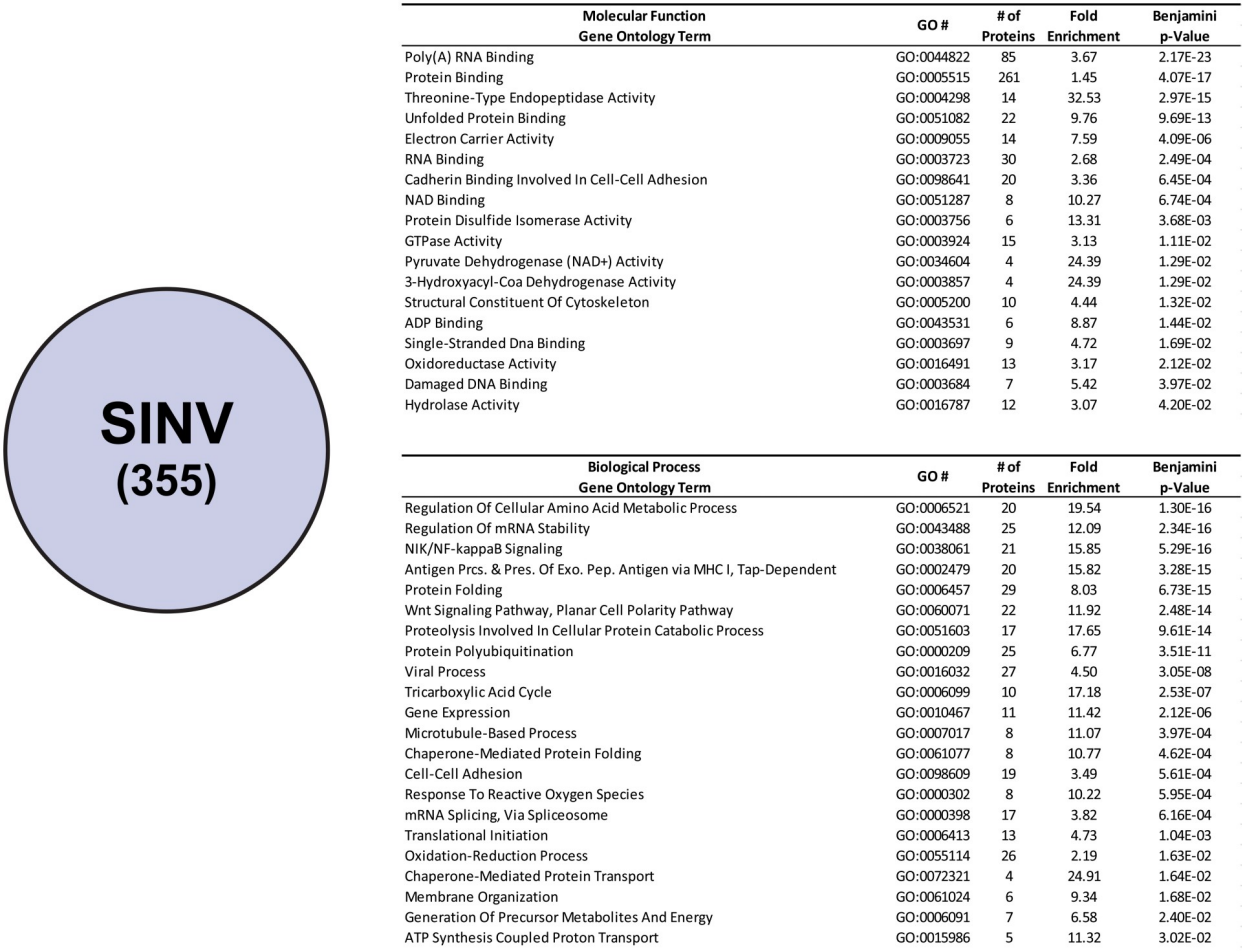
Ontological enrichment analysis of SINV CLAMP-identified interactants. The host factors specific to SINV, as determined by subtractive analysis of the Mock CLAMP data set from the SINV CLAMP data set. Shown are the enriched Molecular Function and Biological Process ontological groups that were statistically enriched with a p-Value of <0.05, as determined via Benjamini-Hochberg procedure. Specific values represent the ontological term name, GO number, number of individual proteins per group, fold-enrichment (relative to the Homo sapiens background data set), and a false discovery rate corrected p-Value, as indicated above.

As shown in Fig 5, ontological assessment of the 355 CLAMP-identified SINV interactants reveals that proteins with the molecular function of Poly(A) RNA Binding (GO:0044822) are enriched to a highly statistically significant degree, with an adjusted p-Value of 2.12x10^-23^. Nonetheless, despite the high statistical significance associated with this enrichment, the overall fold-enrichment is lackluster (~3.7-fold) compared to other enriched Molecular Functions. The mild fold-enrichment value for this ontological group is more than likely due to the large proportion of host proteins which populate this particular group, resulting in a high expected value. The other highly statistically significant Molecular Function ontological groups include Protein Binding (GO:0005515), Threonine-Type Endopeptidase Activity (GO:0004298), and Unfolded Protein Binding (GO:0051082). Of the previously named Molecular Function ontological groups the Threonine-Type Endopeptidase Activity exhibited the highest fold enrichment relative to expected (~32.5-fold). Other Molecular Function ontological groups are comparatively highly enriched, albeit with low statistical significance, such as Pyruvate Dehydrogenase (NAD+) Activity (GO:0034604), and 3-Hydroxyacyl-CoA Dehydrogenase Activity (GO:0003857).

Analyses of the SINV CLAMP-identified interactants in regards to Biological Process ontology also yields insight into the molecular interactions of the SINV viral RNAs during infection. Biological Process ontological groups that are greatly enriched and highly statistically significant include Regulation of Cellular Amino Acid Metabolic Process (GO:0006521), Regulation of mRNA Stability (GO:0043488), NIK/NF-kappaB Signaling (GO:0038061), and Antigen Processing and Presentation of Exogenous Peptide Antigen via MHC I, Tap-Dependent (GO:0002479).

#### Ontological analyses of CHIKV CLAMP-identified interactants

**Fig 6:**
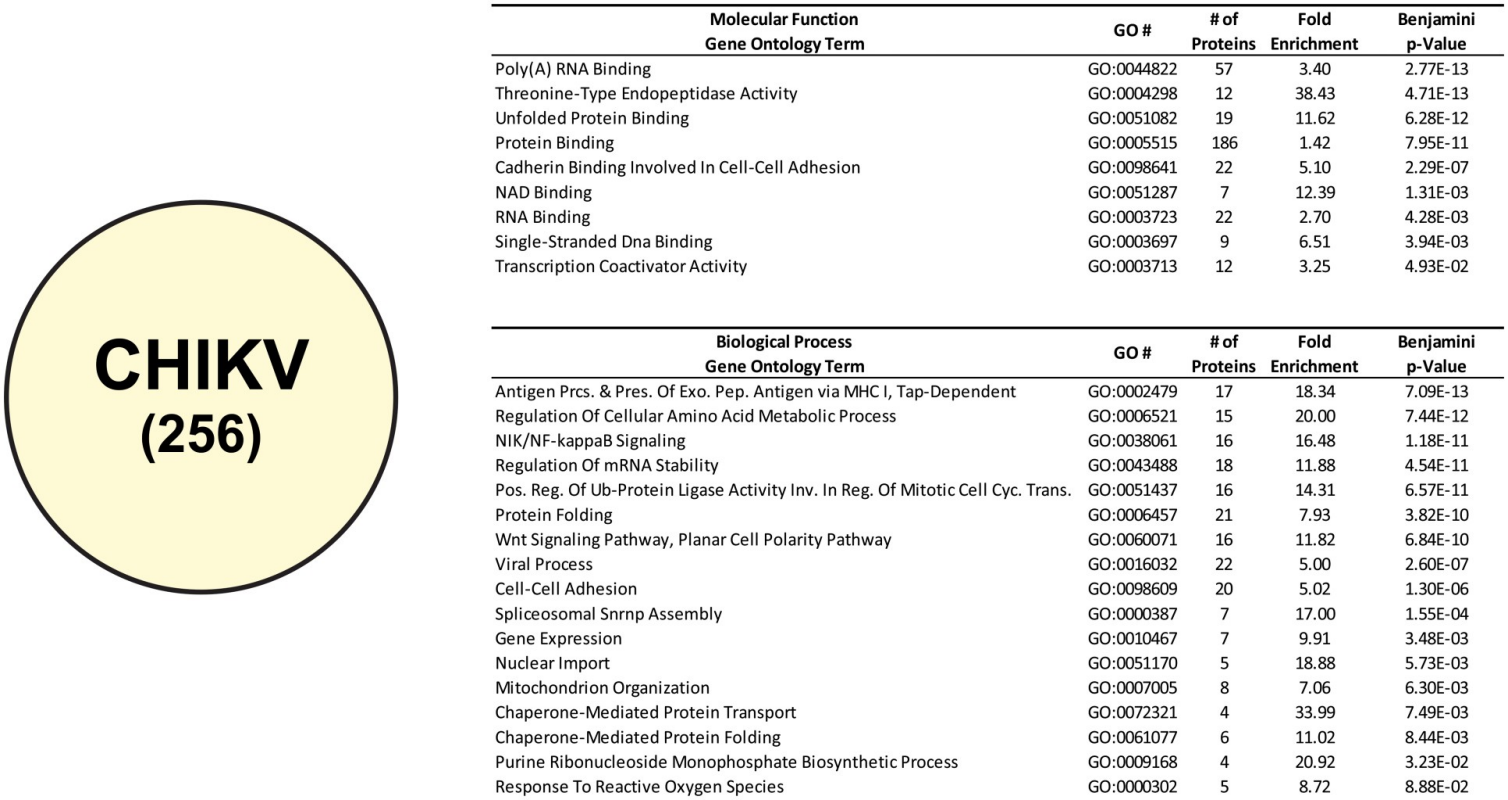
Ontological enrichment analysis of CHIKV CLAMP-identified interactants. The host factors specific to CHIKV, as determined by subtractive analysis of the Mock CLAMP data set from the CHIKV CLAMP data set. Shown are the enriched Molecular Function and Biological Process ontological groups that were statistically enriched with a p-Value of <0.05, as determined via Benjamini-Hochberg procedure. Data is presented identically as described for Fig 3.

Similar analyses of the 256 CLAMP-identified CHIKV viral RNA interactants also reveals that several Molecular Function ontological groups are enriched (Fig 6). In fact, the top five Molecular Function ontological groups were identical to those identified for SINV. Thus, the leading Molecular Function ontological group, in terms of adjusted statistical significance, was Poly(A) RNA Binding (GO:0044822). In addition to the similarity in regards to statistical significance, the relative fold-enrichments for the top five Molecular Function ontological groups were also similar to those observed for SINV.

Nonetheless, despite the high degree of congruence between the SINV and CHIKV Molecular Function ontological enrichments, the enriched Biological Process ontological groups diverged from SINV to a greater degree. In particular, the ordering of the Biological Process ontological groups, in regards to statistical significance, changed between the two analyses. Notable changes include the Regulation of mRNA Stability (GO:0043488) group dropping in rank from the second to the fourth most statistically enriched group.

#### Ontological analyses of VEEV CLAMP-identified interactants

**Fig 7:**
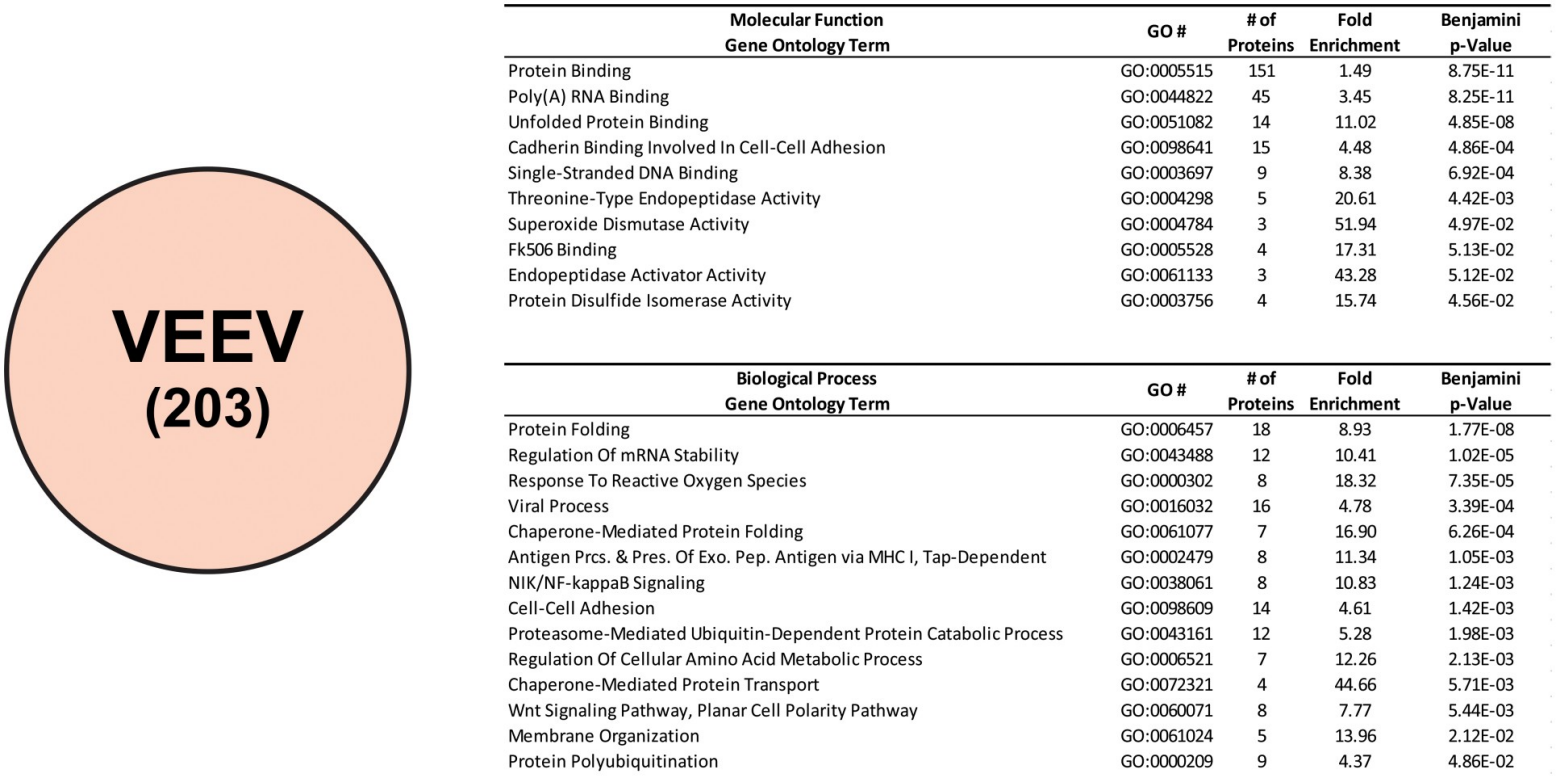
Ontological enrichment analysis of VEEV CLAMP-identified interactants. The host factors specific to VEEV, as determined by subtractive analysis of the Mock CLAMP data set from the VEEV CLAMP data set. Shown are the enriched Molecular Function and Biological Process ontological groups that were statistically enriched with a p-Value of <0.05, as determined via Benjamini-Hochberg procedure. Data is presented identically as described for Fig 3.

Ontological assessment of the CLAMP-identified VEEV interactants results in an overall profile similar to that described above for SINV and CHIKV. However, there are notable differences between the enriched VEEV ontological groups and those detailed above, (Fig 7). The Molecular Function ontological group Protein Binding (GO:0005515) has displaced Poly(A) RNA Binding (GO:0044822) for the top position in regards to statistical significance; however, the difference in magnitude between the two ontological groups is, more or less, negligible. In addition, the Cadherin Binding Involved in Cell-Cell Adhesion (GO:0098641), and Single-Stranded DNA Binding (GO:0003697) are within the top five enriched Molecular Function ontological groups.

The Biological Process ontological groups that were enriched during the analyses of the VEEV CLAMP-identified interactants were also different from those identified for SINV and CHIKV. This is illustrated by the presence of the Biological Process ontological group Response to Reactive Oxygen Species (GO:0000302) amongst the top enriched groups. The primary exception to this generalization is that the Regulation of mRNA Stability (GO:0043488) group is still highly enriched. It should be noted that the statistical significance of the enriched VEEV Biological Process ontological groups are poorer in magnitude relative to those of SINV and CHIKV.

### Comparative analyses of alphaviral CLAMP-identified interactants

While the above ontological analyses of the individual CLAMP-identified interactant data sets reveals the enrichment of particular biological processes for a given alphavirus, through comparative analyses common interactants and ontologically enriched groups may be identified. Despite an exhaustive comparative analysis process, for the sake of brevity only the comparative analysis of all three alphavirus species will be described in detail. Nonetheless, comparisons between the individual pairs of alphaviruses may be found in the Supplemental Data files (S1 Table) accompanying this manuscript.

**Fig 8:**
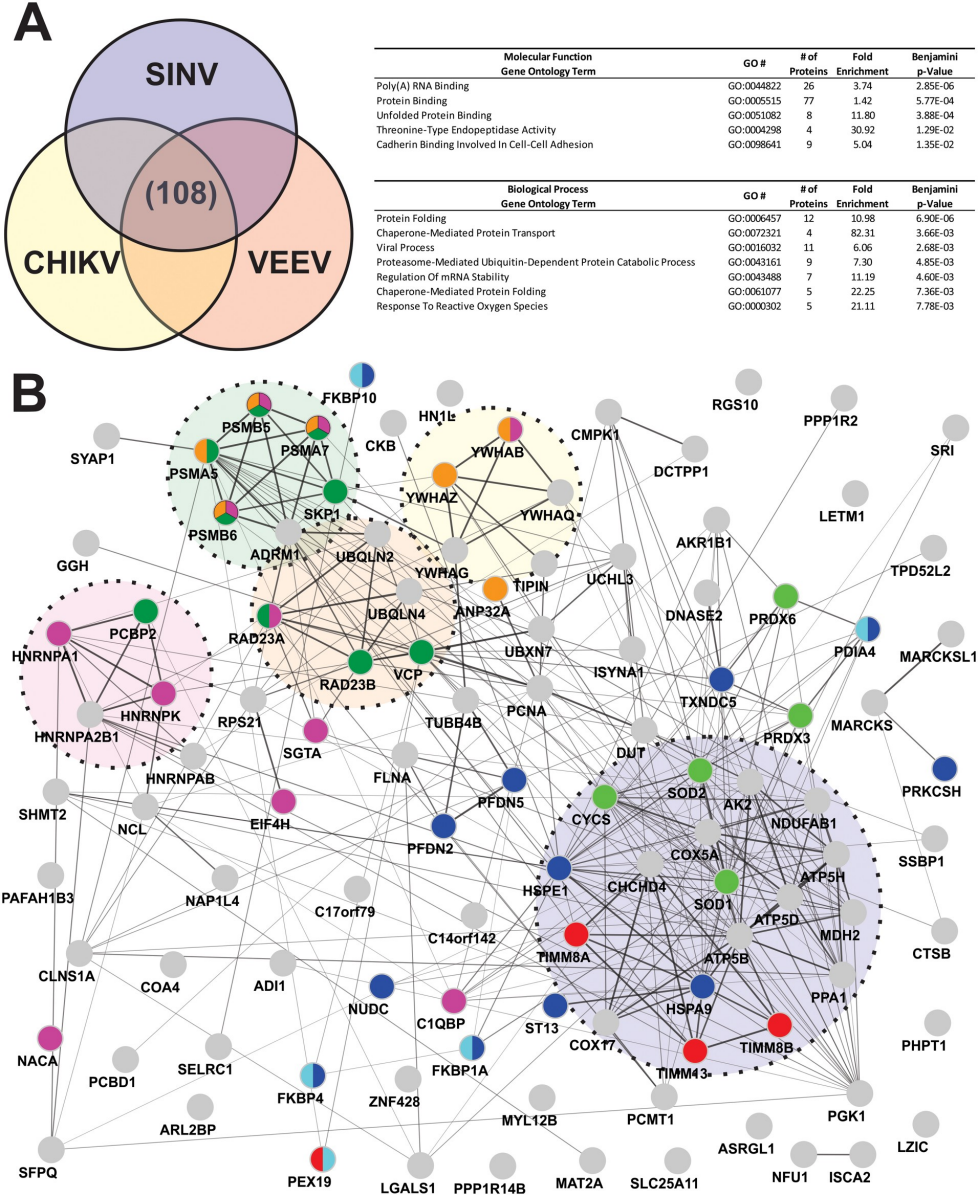
Comparative analysis of SINV, CHIKV, and VEEV CLAMP-identified interactants and ontological assessment of common interactants. (**a**) Comparative analysis of the specific SINV, CHIKV, and VEEV CLAMP-identified interactants reveals 108 host factors in common between the three alphavirus species. Shown are the enriched Molecular Function and Biological Process ontological groups that were statistically enriched with a p-Value of <0.05, as determined via Benjamini-Hochberg procedure. Data is presented identically as described for Fig 5. (**b**) A force-directed interaction map developed via the STRINGs database for Protein:Protein interactions [38, 39]. The weight of the line between two nodes is indicative of the relative strength of the interaction, with a minimum interaction score of 0.40. Individual nodes are color-coded based on which Biological Process ontological groups they belong to- Dark Blue = Protein Folding; Red = Chaperone Mediated Protein Transport; Magenta = Viral Process; Dark Green = Proteasome-Mediated Ubiquitin-Dependent Protein Catabolic Process; Orange = Regulation of mRNA Stability; Light Blue = Chaperone-mediated Protein Folding; and Light Green = Response to Reactive Oxygen Species. Individual protein species which are not categorized into a Biological Process ontological group are colored gray. Interaction Clusters (ICs) are encircled, and color coded as follows- Light Blue = IC1; Seafoam Green = IC2; Tangerine = IC3; Pink = IC4; and Canary Yellow = IC5.

As shown in Fig 8, comparative analysis of the CLAMP-identified interactants of SINV, CHIKV, and VEEV reveals that 108 host factors were identified as common. A comprehensive list identifying the common alphaviral interactants and analyses may be found in the supplemental data accompanying (S1 and S2 Tables) this manuscript. Ontological analyses of the common alphaviral CLAMP-identified interactants provides further insight into the molecular interactions of the alphaviral RNAs during infection (Fig 8A). For both the Molecular Function and Biological Process ontological analyses, the magnitude of statistical significance was pointedly poorer than the virus specific analyses.

The enriched Molecular Function ontological groups of the common alphaviral interactants were Poly(A) RNA Binding (GO:0044822), Protein Binding (GO:0005515), Unfolded Protein Binding (GO:0051082) Threonine-Type Endopeptidase Activity (GO:0004298), and Cadherin Binding Involved in Cell-Cell Adhesion (GO:0098641). As with the previous ontological analyses of molecular function, the ontological group with the highest fold-enrichment relative to expected was Threonine-Type Endopeptidase Activity (GO:0004298).

Biological Process ontological analyses indicates that the Protein Folding (GO:0006457), Chaperone-Mediated Protein Transport (GO:0072321), Viral Process (GO:0016032), Proteasome-Mediated Ubiquitin-Dependent Protein Catabolic Process (GO:0043161), Regulation of mRNA Stability (GO:0043488), Chaperone-Mediated Protein Folding (GO:0061077), and Response to Reactive Oxygen Species (GO:0000302) ontological groups were enriched to a statistically significant degree relative to background. Interestingly, the ontological group with the highest enrichment was the Chaperone-Mediated Protein Transport (GO:0072321), which while present in all data sets, was not a leading enrichment group in any of the individual analyses.

Interaction mapping of the CLAMP-identified common interactants indicates significant interconnectivity. As shown in Fig 8B, the interaction map generated by STRINGs analysis indicates a high degree of interconnectivity within, and between, the Biological Process ontologically enriched categories [38, 39]. Grouping analyses of the interactants indicates the presence of at least 5 clusters based on interactions strengths. The largest STRING interaction group, Interaction Cluster 1 (IC1), consists of proteins associated with mitochondria, in particular proteins involved in mitochondrial transport and the cellular energy generating apparatus. The second largest interaction cluster, IC2, is extensively populated by members of the cellular proteasome complex. The proteins of the cellular proteasome are Threonine-type endopeptidases, which explains the consistent enrichment of the GO:0004298 Molecular Function ontological group. The final 3 interaction clusters are all similar in regards to size and relative interaction strength. The members of IC3 are related to IC2, but were distinct enough to merit their own cluster on the basis of underlying molecular function. Indeed, IC3 consists of proteasome associated proteins, however, unlike IC2 the members of IC3 lack peptidase activity, rather IC3 consists of ubiquitin related proteins. IC4 is a cluster of ribonucleoprotein complex proteins involved in the regulation of mRNA splicing. Finally, IC5 is a collection of interrelated 14-3-3 adaptor proteins.

### Validation of select CLAMP identified common interactants

To verify that the CLAMP assay enables the detection and identification of conserved host / pathogen RNA:Protein interactions, we conducted a series of quantitative immunoprecipitations. As our lab has an avowed interest in the biological roles of host RNA-binding proteins on viral RNA functions during infection, we focused our efforts on the host hnRNP K, hnRNP A1, and ANP32a proteins. The host hnRNP K protein was selected as it has been a subject of previous study by the Hardy and Sokoloski research groups, and thus is a topic of ongoing research interest. Similarly, the hnRNP A1 protein was selected as it has been previously identified as an interactant for several alphaviruses, however has not yet been directly linked to either the viral RNAs of CHIKV or VEEV. Unlike the other two selected proteins, the host ANP32a protein, also known as PHAPI, is a truly novel putative interactant for SINV, CHIKV, and VEEV.

**Fig 9:**
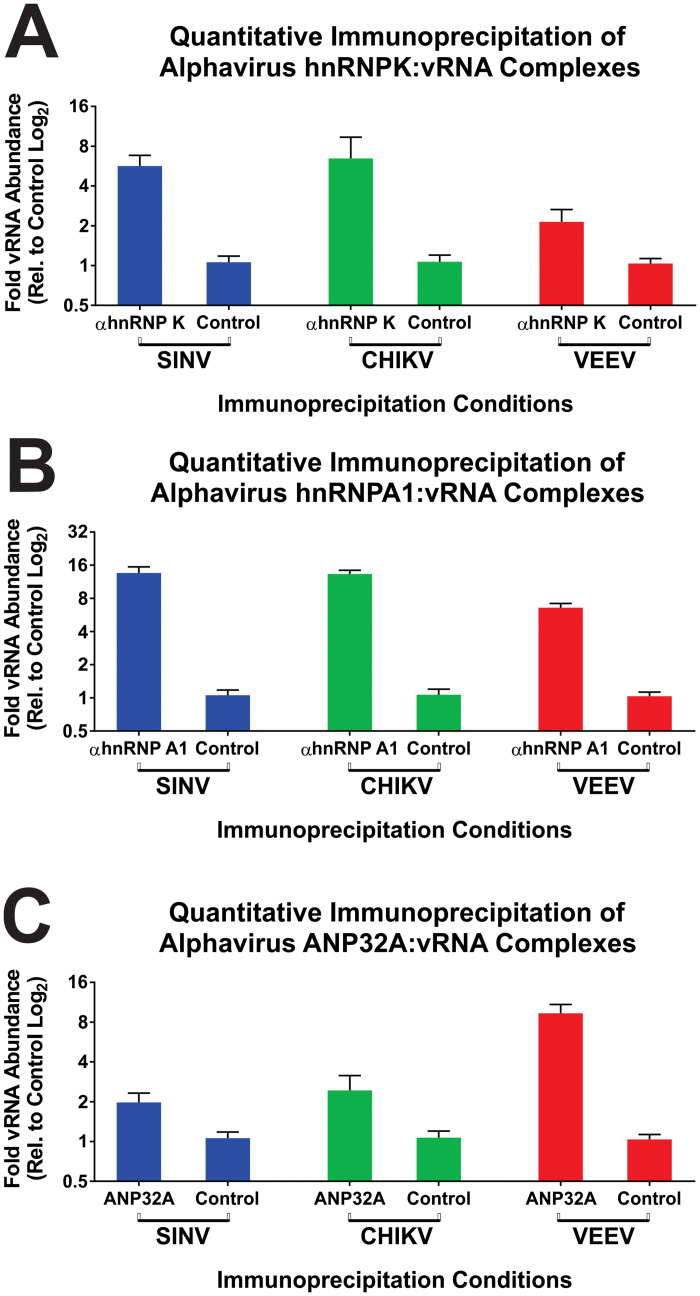
The validation of select CLAMP identified common interactants. 293HEK cells were infected with SINV, CHIKV, or VEEV at an MOI of 10 PFU/Cell and processed as described in the materials and methods. Briefly, the infected cells were cross-linked prior to the generation of whole cell lysates. After clarification via centrifugation the cross-linked lysates were immunoprecipitated with either anti-hnRNP K (Panel A), anti-hnRNP A1 (Panel B), or anti-ANP32a antibodies (Panel C). Nonspecific control immunoprecipitations utilizing an anti-IL1 antibody were conducted in parallel. After immunoprecipitation the cross-links were reversed, and the co-immunoprecipitated RNAs were used as input materials for the synthesis of random hexamer primed cDNAs. The positive sense viral RNA species of SINV, CHIKV, and VEEV were then assayed using qRT-PCR and plotted as the fold abundance of the viral RNA relative to the control immunoprecipitations. The quantitative data shown is the means of three independent biological replicates, and the error bar represents the standard deviation of the means.

As demonstrated by the data in Fig 9, immunoprecipitation of cross-linked RNA:Protein complexes from SINV, CHIKV, and VEEV infected 293HEK cells resulted in the enriched co-purification of the positive-sense RNAs in the presence of anti-hnRNP K, anti-hnRNP A1, and anti-ANP32a antibodies relative to immunoprecipitations utilizing a nonspecific control antibody. Notably, with the exception of perhaps hnRNP A1, the magnitudes to which the viral RNAs co-immunoprecipitated varied. The precise cause and implications of this are unclear. One potential explanation for this phenomenon is that although the three alphaviruses do in fact share a protein as a common interactant, the three viruses may exhibit different affinities for the host factor by utilizing alternative interaction motifs.

## Discussion

As described above, CLAMP analysis has revealed a broad network of host / pathogen interactions for SINV, CHIKV, and VEEV. Ontological analyses of Molecular Function reveal highly significant enrichments of RNA-associated proteins for each tested alphavirus, confirming the capacity of the CLAMP strategy to lead the discovery of Protein:vRNA interactions. Furthermore, the nature of the cross-linking method used enables the identification of distal interactions via the formation of Protein:Protein interactions proximal to, or in partnership with, the Protein:vRNA interactions during infection. These secondary interactions are likely represented by the enrichment of proteins associated with non-RNA binding activities, including host proteases, ubiquitin related proteins, and host proteins central to mitochondrial activity.

Comparative assessments of the alphaviral CLAMP-identified interactants yields several interesting insights into alphaviral host / pathogen interactions. Foremost, there are numerous conserved proteins associated with the Poly(A) RNA Binding Molecular Function ontological group, including several RNA-binding proteins. Specifically, a number of hnRNP proteins, such as hnRNP A1, E2 (PCBP2), K, A2B1, and AB, are readily identified as conserved interactants. These hnRNP proteins are largely responsible for the statistical enrichment of the Regulation of mRNA Stability Biological Process ontological group. Whether or not these proteins function in relation to the evasion of the host RNA decay machinery is yet to be exhaustively characterized. Moreover, previous studies have indicated that the functions of the hnRNP proteins during alphaviral infection are highly complex, and may not be unified in respect to function across the members of the genus [31, 42]. In addition, the cellular La protein (SSBP1), which has been previously described to interact with the negative sense RNAs of SINV [27–29], is identified as a common interactant. Several of the aforementioned RNA-binding proteins have been previously identified as interactants with one- or more alphaviruses; however, the data documented here represents the first time in which they have been identified as conserved across SINV, CHIKV, and VEEV.

Another notable cluster of conserved interactions centers around proteins associated with the host proteasome. Chemical inhibitors of the proteasome have been previously demonstrated to impact VEEV infection; however, the data shown above infer that other alphaviruses may be similarly affected by MG132 and Bortezomib [43]. The precise function of the proteasome during alphaviral infection remains unknown, and represents an interesting subject for further exploration. In addition to the proteosomal proteins, a cluster of ubiquitin associated proteins was identified as common to SINV, CHIKV, and VEEV. The implications of these interactions aren’t clear, but they are likely related to the functional activities of the proteasome.

A further novel cluster of host proteins are the 14-3-3 adaptor proteins YWHAZ (14-3-3 zeta), YWHAB (beta), YWHAQ (tau), and YWHAG (gamma). Normal cellular functions of the 14-3-3 adaptor proteins involve their respective binding to cell signaling proteins, including kinases, phosphatases, and transmembrane receptor proteins, as reviewed by [44–46]. Furthermore, reports have indicated increased 14-3-3 protein levels in the synovial space during joint tissue injury; however, whether or not this is observed during alphaviral infection is unknown [47]. Several peptide and small molecule inhibitors of 14-3-3 adaptor proteins have been described [48]. Nonetheless, the extent to which these compounds affect alphaviral infection is unknown.

Despite causing notably different diseases, the alphaviruses assayed with the CLAMP method in these studies all share a similar underlying molecular life cycle. Thus, despite their capacity to result in clinically different illnesses, the targeting of a common interactant may have wide ranged therapeutic potential by disrupting or preventing the successful completion of the viral life cycle. Interfering with viral replication can be reasonably expected to alleviate viral disease regardless as to whether the infection resulted in encephalitic or arthritic disease.

### Strengths, limitations, caveats, and potential applications of the CLAMP assay

As demonstrated by our previous report and the data reported above, the CLAMP assay is a robust discovery approach capable of identifying novel host / pathogen interactions. A major strength of the CLAMP method is that it is highly adaptable, requiring only that the target viral transcript(s) be capable of being selectively labeled with the 4SU nucleotide analogue. As cellular transcription can be inhibited through a number of available small molecule inhibitors, any number of RNA viruses could be assessed using the CLAMP method. Similarly, any number of host cell types or systems could be assessed to identify host cell type or host species specific interactions. Complementary CLAMP assessments of individual viral strains, such as those which are virulent and avirulent, could also potentially reveal the existence of any differential host factor interactions. While we have not directly expanded the use of the CLAMP assay along these lines of study, the CLAMP methodology could be readily used in such manners to further define the molecular biology of viral infections. Thus, the future application of the CLAMP approach to other viruses and hosts will undoubtedly lead to the identification of previously undetected RNA:Protein interactions with important biological roles to infection.

In addition to the flexibility of the CLAMP assay in regards to the virus and host, further adaptation of the assay could enable the assessment of RNA:Protein complexes with respect to time. Although not directly evaluated in this study, the CLAMP assay could be modified to assess whether or not temporal differences in RNA:Protein complexes were present by altering the timing and duration of the 4SU labeling period prior to cross-linking event. For instance, shortening the labeling period and altering the initiation of the cross-linking event relative to the labeling period in a serial manner (as per a standard pulse-chase experiment) would allow the sequential analysis of RNA:Protein interactions for a given population of viral RNAs with respect to time. Care should be taken when designing such experiments to ensure that any potential differences in viral RNA kinetics are accounted for. Similarly, the recovery of the viral RNAs should be assessed to ensure that appropriate comparisons are being made. These notions are also true when the CLAMP data of several interrelated viruses are comparatively assessed to identify common interactants, as is reported here. Significant differences in regards to viral RNA synthesis kinetics or recoveries could confound data interpretation and lead to the exclusion of potential host interactions. As demonstrated by the data reported in Figs 2 and 3 this is not a concern in these studies, but it is essential that any application of the CLAMP assay be well characterized to ensure proper comparisons are being made. Furthermore, it should be noted that altering the length of the labeling period may also impact the overall magnitude of recovery of the viral RNAs, which could inadvertently limit the “sight” of the CLAMP assay.

While the clear focus of our CLAMP studies to date have been the identification of novel host / pathogen interactions, the CLAMP assay could also be utilized to probe the interactions of viral proteins with the viral RNAs during infection. For the alphaviruses these would expected to be the proteins of the viral replication machinery (consisting of nsPs 1 through 4), and the viral capsid proteins. Indeed elements of the viral replication machinery were identified during the CLAMP assays described above; however, as the rigorous assessment of the RNA synthetic complexes were not the focus of our studies they were ignored in our analyses. Nonetheless, the CLAMP assay could be utilized to directly probe the interactions of the viral RNAs with viral proteins.

A notable caveat of this approach, and truly all discovery approaches, lies with the inherent limitations of protein detection via mass spectrometry. This is especially true for comparative analyses such as those described above, where the absence of a particular interactant from a data set may or may not be due to the genuine absence of the protein interaction, but also may be due to a lack of detection during mass spectrometry. These instances may arise from low target abundances, poor recoveries, and other intrinsic properties of the protein itself (such as poor ionization during mass spectrometry). Hence it is important that discovery approaches, such as the CLAMP assay, be recognized as tools to identify potential interactions, and not the rigorous exclusion of them.

## Conclusions

To conclude, comparative analysis of the host / pathogen interactions of SINV, CHIKV, and VEEV have identified a number of conserved interactants. While the functional aspects of these interactions are largely unknown, they represent novel targets for further biological exploration. Moreover, these studies underscore the importance of Protein:RNA discovery methods, and validate the robust potential of the CLAMP assay.

## Supporting information

S1 Dataset Raw CLAMP mass spectrometry data. (ZIP)

S1 Table Individual CLAMP-interactant lists and pairwise comparisons of alphaviral CLAMP-interactant list. (XLSX)

S2 Table Ontological analyses. (XLSX)
